# Updating the Endemicity Map of Soil-Transmitted Helminthiasis in Ten Local Government Areas of Ondo State, Southwestern Nigeria

**DOI:** 10.3390/tropicalmed11010024

**Published:** 2026-01-14

**Authors:** Uwem F. Ekpo, Jacob Solomon, Hammed O. Mogaji, Francisca O. Olamiju, Fajana Oyinlola, Ijeoma Achu, Olanike O. Oladipupo, Alice Y. Kehinde, Imaobong O. Umah, Fatai Oyediran, Moses Aderogba, Louise K. Makau-Barasa

**Affiliations:** 1 Centre for Parasite Epidemiology and Control, Department of Zoology, Akwa Ibom State University, PMB 1167, Uyo 520101, Nigeria; 2 Department of Pure and Applied Zoology, Federal University of Agriculture, Abeokuta 110001, Nigeria; 3 Mission to Save the Helpless, Lagos 100001, Nigeria; olamijufo@mitosath.org (F.O.O.); fajanao@mitosath.org (F.O.); achuic@mitosath.org (I.A.); 4 Neglected Tropical Diseases Program Unit, Department of Public Health, Federal Ministry of Health, Abuja 900001, Nigeria; owukpa2000@yahoo.com (J.S.); imaumah@yahoo.com (I.O.U.); fatai_oyediran@yahoo.com (F.O.); 5 Public Health Program, Department of Behavioural and Applied Social Sciences, Marian University, Indianapolis, IN 46222, USA; 6 Neglected Tropical Diseases Program Unit, Department of Public Health, Ondo State Ministry of Health, Akure 340001, Nigeria; olanikeoladipupo1@gmail.com; 7 Neglected Tropical Diseases Program Unit, Department of Public Health, Federal Ministry of Health, Akure 340001, Nigeria; kehindealice70@gmail.com; 8 Ending Neglected Diseases Fund, New York, NY 10016, USA; maderogba@endfund.org (M.A.); lmakau-barasa@endfund.org (L.K.M.-B.)

**Keywords:** soil-transmitted helminthiasis, endemicity, impact, PC, Ondo State, Nigeria

## Abstract

As Nigeria advances toward the elimination of soil-transmitted helminthiasis (STH), updated endemicity maps are essential for guiding programmatic decisions. A cross-sectional study was conducted to update the STH endemicity maps in ten local government areas (LGAs) of Ondo State from July to August 2024. LGAs were stratified into three categories (C1–C3) based on the history of preventive chemotherapy (PC), with C1 being endemic LGAs with ≥5 effective rounds of PC, C2 being endemic LGAs with <5 effective rounds of PC, and C3 being low-endemicity (STH prevalence <20%; PC not required). A total of 4507 school-aged children (5–14 years) from 151 systematically selected communities (15 per LGA) provided fresh stool samples to assess the prevalence and intensity of STH. Stool samples were examined using the Kato-Katz technique. Prevalence of STH was aggregated at the LGA level and compared with World Health Organization thresholds. In the first category (C1), the baseline prevalence was reduced significantly by 60–96%, with specific prevalence in Akoko Southwest (from 28.2% to 0.4%, Risk Ratio (RR): 0.01), Akure North (from 39% to 1.5%, RR = 0.04), Ifedore (from 25% to 2.5%, RR = 0.10), and Ondo East (from 45.2% to 8.2%, RR = 0.18). In the second category (C2), the baseline was reduced significantly by 66–100%, with Akure South (from 29% to 1.2%, RR = 0.04), Ose (from 20% to 2.2%, RR = 0.11), Owo (~100% reduction), and Odigbo (38% to 12.8%, RR = 0.34). In the C3 LGAs, infection was significantly below the baseline threshold, with Akoko Northwest (5.2% to 0.9%, RR = 0.17) and Idanre (from 14.2% to 1.8%, RR = 0.13). Overall, significant reductions in STH prevalence were observed across the surveyed LGAs, with risk ratios ranging from 0.04 to 0.40. These findings updated the endemicity map for the ten LGAs in Ondo State, demonstrating significant progress toward STH elimination following PC implementation.

## 1. Introduction

More than 1.5 billion people are at risk of soil-transmitted helminthiasis (STH), with the majority residing in sub-Saharan Africa [1,2,3]. STH continues to be a neglected tropical disease of public health importance and is targeted for control by 2030 [2,3]. *Ascaris lumbricoides* (Linnaeus, 1758), *Trichuris trichiura* (Linnaeus, 1771), and hookworms (*Necator americanus* (Stiles, 1902) and *Ancylostoma duodenale* (Dubini, 1843) are the four common parasitic nematodes causing STH and are intensively transmitted in areas where inadequate sanitation, unsafe water, and poor hygiene practices persist [4,5,6,7]. School-aged children (SAC) are particularly vulnerable and have been the target of existing preventive chemotherapy control programs, which involve the mass administration of albendazole or mebendazole (MDA) without prior diagnosis in endemic areas [3,8,9]. Over the past decade, the World Health Organization has supported PC programs by distributing over 600 million donated anthelmintic medicines [10].

The implementation of PC programs is guided by endemicity thresholds at the implementing unit (IU), usually the local government areas (LGAs), districts, or provinces [9]. For instance, biannual PC (twice-per-year mass administration) is implemented in areas where prevalence exceeds 50%, annual PC is implemented in areas where prevalence is between 20 and 49.9%, and clinical case management is implemented when prevalence is below 20% [9,10]. As part of programmatic guidelines, the PC program is considered only effective on the parasite population when more than 75% of the targeted school-aged children within an IU receive treatment over a sustained period of 5–6 years [10].

PC programs for STH are expensive and do not need to continue when the endemic threshold falls below 20% [10]. Updating the STH endemicity map through impact assessments is therefore recommended after 5–6 years of effective implementation to determine program effectiveness, optimize resource allocation, and ascertain progress toward elimination [10]. Nigeria remains the most highly endemic country for STH in sub-Saharan Africa [11,12]. With extensive PC campaigns implemented over five years in Ondo State [13], it is expected that formerly endemic areas may now have reduced transmission. A recent evaluation of the endemicity of STH in three LGAs in Ondo State highlighted the need for an update on the endemicity profile in other LGAs [13]. Hence, in this study, we evaluated STH prevalence and intensity across ten LGAs with varying PC exposure histories to evaluate the impact of the PC program and update the state STH endemicity map.

## 2. Materials and Methods

### 2.1. Study Area

Ondo State is in the southwest zone of Nigeria, comprising 18 LGAs and 206 wards, with a population exceeding three million. The tropical climate and diverse vegetation zones (freshwater swamps, rainforests, and Guinea savannah) influence helminth transmission dynamics. The state contains numerous rivers and ponds that support agriculture, fishing, and recreational activities, which increase the risk of water-related exposure. The state has a population of 5,540,403, of which 1,551,313 are SAC [13].

### 2.2. Study Design and Sampling

We employed a cross-sectional sampling design and collected stool samples and questionnaire data from school-aged children (SAC) between July and August of 2024. SAC were sampled across 151 systematically selected communities (15 per LGA) in ten LGAs using methods previously described [13]. The sample size followed WHO guidelines of recruiting 30 SAC per community [13].

### 2.3. Selection of Study LGAs

**Figure 1:**
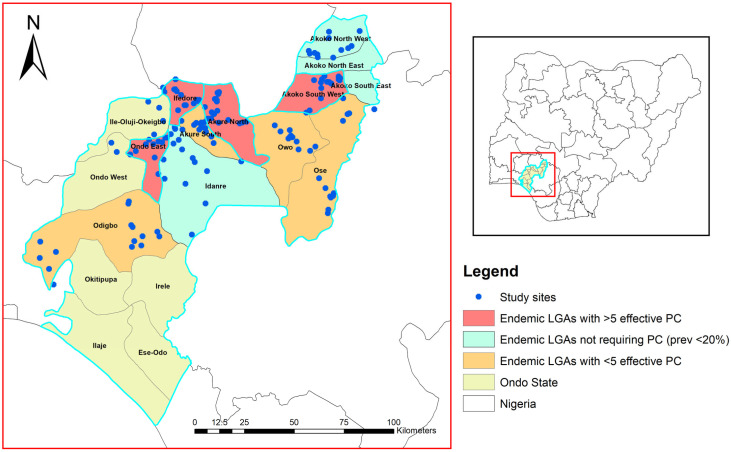
Map of Ondo State with Nigeria as an inset, showing the study sites, different endemicity categories of the LGAs and treatment history. The highlighted LGAs were selected for this study. Legend: This map was created using primary data collected by the authors and developed in ArcGIS v10.3. The shapefiles for Nigeria, including boundary polygons and water bodies across all administrative levels, were sourced from publicly accessible databases available at Humanitarian Data Exchange (https://data.humdata.org/). The map is not copyrighted, and the authors grant permission for its re-use.

Baseline epidemiological mapping for STH in Ondo revealed that 12 LGAs had endemicity between 20 and 50%, and 2 LGAs had endemicity >50% [14]. These endemic LGAs benefited from an average of nine rounds of PC with albendazole. Hence, for this study, we selected LGAs based on their baseline prevalence and PC history. We stratified into three categories (C1-C3), with C1 being endemic LGAs with ≥ 5 effective rounds of PC (n=9), C2 being endemic LGAs with < 5 effective rounds of PC (n=5), and C3 being low endemicity (STH prevalence <20%; PC not required, n=4). In C1, three LGAs (Ese-Odo, Ile-Oluji, and Irele) had been previously assessed, with findings published elsewhere [13], and were excluded from the sampling frame. Of the remaining six LGAs, four (Akoko Southwest, Akure North, Ifedore, and Ondo East) were selected for the study. In C2, four LGAs (Akure South, Ose, Owo, and Odigbo) were selected, and in C3, two LGAs (Idanre and Akoko Northwest) were selected. The selection of LGAs across all categories was purposive, guided by the need to include areas co-endemic for schistosomiasis, which allowed for an integrated assessment of both diseases and ensured optimal use of available resources (Figure 1).

### 2.4. Selection of Study Sites

Study sites were selected systematically. Briefly, in each LGA, names of communities were sourced from LGA coordinators and were listed by ward or subdistrict and re-arranged based on proximity. In each ward, one community was randomly selected by using paper ballots. This approach ensures that each sub-district has an unbiased and equal chance of selection while also providing adequate geographic representation across the district and its sub-districts. Study sites that were inaccessible or challenged by insecurity were replaced using paper ballots (Figure 1).

### 2.5. Stool Collection and Processing

Fresh stool samples were collected from the participating school children with the help of their parents and teachers. A pre-labeled sterile specimen bottle was provided and retrieved from the SAC after 1 h of distribution and specimens. Participants were provided with applicator sticks, plain sheets of paper, tissue paper, and soap to assist with stool collection. Samples were transported in iceboxes to the laboratory at the Ondo State College of Health Technology, Akure, for processing using the Kato-Katz technique within two hours of collection. A single thick smear was prepared from each sample and allowed to clear for 30 min. The smears were examined microscopically for Ascaris, *Trichuris,* or hookworm parasite eggs. For quality assurance, each slide was re-examined by a second microscopist, and the egg counts were verified. A participant was classified as positive if at least one egg from any target parasite was detected per slide.

### 2.6. Questionnaire Administration

We utilized closed-ended electronic questionnaires deployed on Kobocollect platform to collect demographic data, including age and sex, and document parasitological results from examined stool samples. Interviews were conducted in either Yoruba or English and held confidentially, with a legal guardian or parent present when necessary.

### 2.7. Data Analysis

Data were analyzed using R Studio (version 4.3.2). The overall infection status (i.e., any STH infection) and species-specific prevalence were determined using the WHO threshold (<2%, 2% to <10%, 10% to <20%, and ≥20%). Intensity was expressed as eggs per gram (EPG), as described previously [13]. Descriptive statistics were used to summarize categorical variables, with a 95% confidence interval computed for proportions. Risk ratios were also used to compute the impact of PC between baseline and endline estimates. Spatial distribution maps of STH across the LGAs were generated using ArcGIS version 10.8.

## 3. Results

### 3.1. Study Population

Table 1 presents a comprehensive profile of the study locations in the LGAs. The coverage within the subunits of the LGAs ranged from 73.3% to 100%, with Akure North being the only LGA to achieve full coverage across its subunits. Other LGAs encountered challenges such as insecurity, which limited their coverage. Despite these challenges, the number of communities sampled exceeded the target (151 vs. 150), as did the total number of participants recruited (4507 vs. the targeted 4500). Although most LGAs surpassed their target sample size, a few recruited fewer participants than anticipated: Akure South (*n* = 421, 93.6%), Ifedore (*n* = 444, 98.7%), and Owo (*n* = 430, 95.6%). Stool and urine samples were collected from 98.7% of the participants. There were no significant differences in recruitment by gender (*p* = 0.063) and age (*p* = 0.061), although slightly more younger children aged 5–9 years (*n* = 2300, 51%) were recruited than those aged 10–15 years (*n* = 2207, 49%) (Table 1).

### 3.2. Prevalence of Soil-Transmitted Helminthiasis Across the LGAs

Table 2 summarizes the prevalence of soil-transmitted helminthiasis (STH) across local government areas (LGAs). By species’ prevalence, *Ascaris lumbricoides was the most predominant*, followed by hookworm and *Trichuris trichiura* (Table 2). For *Ascaris*, endemic LGAs with fewer than 5 effective rounds had a prevalence range between 0 and 7.5%; those with more than 5 effective PC rounds had a prevalence between 0.2 and 6.1%. Low endemic LGAs had a prevalence between 0.4 and 1.1%. For hookworm, endemic LGAs with fewer than 5 effective rounds had a prevalence range between 0.5 and 6.4%; those with more than 5 effective PC rounds had a prevalence between 0 and 2.3%, while low endemic LGAs had a prevalence between 0.4 and 0.7%. For *Trichuris*, endemic LGAs with fewer than 5 effective rounds had a prevalence range between 0 and 0.4%; those with more than 5 effective PC rounds had a prevalence between 0 and 0.2%, while low endemic LGAs had no *Trichuris* parasite.

Across the LGAs surveyed, the prevalence of any soil-transmitted helminth (STH) infection significantly decreased during the impact assessment compared to baseline levels. In the first category (i.e., endemic LGAs with fewer than 5 effective rounds), the baseline prevalence was reduced significantly by 60–96%. In Akoko Southwest, the prevalence of any STH decreased from 28.2% to 0.4%, resulting in a 99% reduction and a risk ratio of 0.01. In Akure North, the prevalence decreased from 39% at baseline to 1.5%, resulting in a 96.2% reduction, with a risk ratio of 0.04. Ifedore experienced a decrease from 25% to 2.5%, representing a 90% reduction, with a risk ratio of 0.10. Ondo East recorded a decrease from 45.2% to 8.2%, resulting in an 81.9% reduction and a risk ratio of 0.18.

In the second category (i.e., endemic LGAs with more than 5 effective rounds), endemicity from baseline was reduced significantly by 66–100%. Akure South experienced a reduction from 29% to 1.2%, leading to a 95.9% decrease and a risk ratio of 0.04. In Ose, the prevalence decreased from 20% to 2.2%, leading to an 89% reduction, with a risk ratio of 0.11. In Owo, no infections were detected during the impact assessments, leading to a 100% reduction. Odigbo showed a reduction from 38% to 12.8%, marking a 66.3% decrease and a risk ratio of 0.34.

In the third category (i.e., low endemic LGAs with no PC history), infections were significantly below the baseline threshold. In Akoko Northwest, there was a significant decrease of 82% from a 5.2% baseline prevalence to 0.9%. In Idanre, there was a reduction from 14.2% at baseline to 1.8%, reflecting an 87.3% decrease and a risk ratio of 0.13. Overall, significant reductions in STH prevalence were observed across the surveyed LGAs, with risk ratios ranging from 0.04 to 0.40 (Table 2). Additionally, there were no moderate or heavy infections in any STH species across the LGAs (Table 3).

### 3.3. Programmatic Interpretation of STH Prevalence Data Across the LGAs

**Figure 2:**
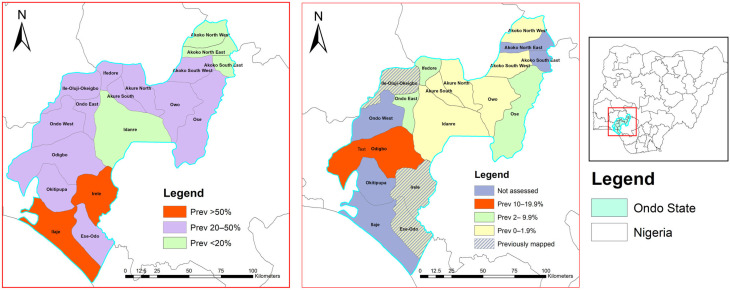
Map of Ondo State showing the endemicity of the LGAs at baseline and impact assessment. Legend: This map was created using primary data collected by the authors and developed in ArcGIS v10.3 software. The shapefiles for Nigeria, including boundary polygons and water bodies across all administrative levels, were sourced from publicly accessible databases available at Humanitarian Data Exchange (https://data.humdata.org/). The map is not copyrighted, and the authors grant permission for its reuse.

Table 4 presents the World Health Organization (WHO) recommendations for MDA based on the varying prevalence of STH. For LGAs with a prevalence rate between 0 and 2%, the recommended action is to conduct MDA during selected events and to establish and maintain surveillance. The LGAs in this category include Owo, which has a prevalence rate of 0%, Idanre at 1.8%, Akoko Southwest at 0.4%, Akure South at 1.2%, Akoko Northwest at 0.9%, and Akure North at 1.5%. These areas are considered low-risk for STH and require careful monitoring to ensure that their prevalence remains low. In the 2–10% prevalence category, the recommendation is to conduct MDA once every two years for five years, along with establishing and maintaining surveillance. The LGAs that fall under this category are Ifedore (2.5 %), Ondo East (8.2 %), and Ose (2.2 %). These areas are recognized as having moderate prevalence, necessitating more frequent interventions to prevent escalation and sustain public health. For LGAs with a prevalence between 10 and 20%, MDA should be conducted once for five years, combined with ongoing surveillance. Odigbo was the only LGA identified in this category, with a prevalence rate of 12.8%. This area presents a greater concern and requires targeted interventions to mitigate health risks effectively (Figure 2).

### 3.4. Elimination Insights for STH Across Ten LGAs

All LGAs met the first (prevalence is less than 20%) and second (prevalence of MHI infection is less than 2%) conditions outlined by the WHO for progressing towards elimination. However, open defecation, which is a proxy for poor WASH access, is still a predominant practice across the LGA (ranging from 8.9 to 60.1%). In addition, MDA programs are still largely targeted at SAC and seldom delivered to WRA during community-based interventions involving the treatment of lymphatic filariasis. Efforts to improve WASH and expand MDA to WRA are essential (Table 5 and Figure 2).

## 4. Discussion

This impact assessment shows substantial progress in STH elimination in Ondo State. LGAs with ≥5 effective PC rounds achieved the greatest reduction, highlighting the value of sustained coverage. LGAs with fewer PC rounds and non-endemic LGAs exhibited significant reductions in STH prevalence. The importance of this assessment is threefold: first, to determine the effectiveness of preventive chemotherapy (PC) in reducing worm burden; second, to prevent misallocation of resources to areas where interventions are no longer necessary, thus optimizing investment in underserved regions; and third, to generate evidence essential for tracking progress and refining PC programs for maximum impact [15]. Across all four endemic LGAs that completed the required PC rounds, the prevalence declined by 81–99%. These findings updated the endemicity map for the ten LGAs in Ondo State, demonstrating significant progress toward STH elimination following PC implementation. Endemic LGAs with fewer than five effective PC rounds recorded reductions of 66–100%. For the lowly endemic LGAs with baseline prevalence below 20% and where no PC is required, there was also a positive decline between 82.7% and 87.3%. Overall, LGAs with more than five years of effective implementation experienced the largest reduction. These findings align with other evidence on the impact of effective PC [13,16]. Infection intensity, a more reliable measure of disease burden, remains very low across all LGAs and meets WHO targets of reducing the prevalence of moderate or heavy intensity of infection to below 2% [15]. The species distribution and prevalences observed in this study are consistent with national patterns, with *Ascaris* being most prevalent, followed by hookworm and *Trichuris* [16,17,18]. *Trichuris* infection has a relatively restricted distribution in most parts of Nigeria, a circumstance that has likely contributed to the overall success of soil-transmitted helminth control programs, given the limited efficacy of albendazole against this species [19]. However, in Odigbo LGA, *Trichuris* infection was detected in 2 of 453 participants examined (0.4%), and this LGA also recorded the lowest overall reduction in STH prevalence (~66%) across the study area. The presence of *Trichuris*, even at low prevalence, may therefore undermine control gains, underscoring the need for intensified interventions—such as improved delivery of albendazole in combination with ivermectin—in areas where *Trichuris* is observed [20].

Programmatically, two LGAs (Akure South-west and Akure North) with 5 rounds of effective PC no longer require MDA, while two others (Ifedore and Ondo East) will need biennial MDA for the next five years. Two LGAs (Owo and Akure South) with less than five required rounds of PC no longer require MDA, while Ose LGA requires biennial and Odigbo LGA requires annual MDA for the next five years. These findings reinforce the importance of completing at least five effective rounds of MDA, especially in LGAs with baseline endemicity [13,15,21].

Achieving STH elimination requires four conditions: (1) reducing overall prevalence to <20%; (2) reducing moderate/heavy infections to <2%; (3) ensuring treatment coverage among women of reproductive age (WRA); and (4) improving access to water, sanitation, and hygiene (WASH), particularly eliminating open defecation. All LGAs met the first two conditions, but progress on WRA coverage and WASH remains limited. Open defecation remains common and threatens the sustainability of gains. Recent evidence from within the area shows that poor hygiene practices—especially not washing hands with soap after defecation—pose the highest infection risk, followed by unimproved latrines and unsafe water sources [13]. Similar findings have been reported in Ethiopia [22], Angola [23] and Kenya [24], where inadequate WASH infrastructure undermined the success of PC outcomes [24,25]. Integrating strong WASH intervention into the PC program is therefore essential for long-term success [26].

From a policy perspective, these results emphasize the continued need for MDA investment, regular impact assessments, and complementary WASH interventions. A major strength of the study is its inclusion of both enrolled and non-enrolled school-aged children, reducing bias inherent in school-based sampling. Although deworming through the Lymphatic Filariasis program—albendazole and ivermectin—may have contributed to observed reductions, this reflects real-world implementation. A key limitation is the absence of baseline WASH, which restricts assessment of WASH-related progress over time.

## 5. Conclusions

Preventive chemotherapy has significantly reduced STH prevalence and intensity across Ondo State. Overall, significant reductions in STH prevalence were observed across the surveyed LGAs, with risk ratios ranging from 0.04 to 0.40. These findings updated the endemicity map for the ten LGAs in Ondo State, demonstrating significant progress toward STH elimination following PC implementation. To maintain progress toward elimination, integrated strategies combining PC, WASH improvement, and expanded coverage for women of reproductive age are essential.

## Figures and Tables

**Table 1:** Profile of study locations and participants.

		Endemic LGAs with ≥ 5 Effective Rounds				Endemic LGAs with ≤ 5 Effective Rounds				Low Endemicity and No PC Required	
		**AKSW**	**AKN**	**IFE**	**ONE**	**AKS**	**OSE**	**OWO**	**ODI**	**IDA**	**AKNW**
**Administrative units**											
Number of sub-units/wards	112	15	12	10	10	11	12	11	11	10	10
Sub-units/wards surveyed	95 (84.8)	11 (73.3)	12 (100)	9 (90.0)	9 (90.0)	9 (81.8)	9 (75.0)	9 (81.8)	9 (81.8)	9 (90.0)	9 (90)
Communities targeted	150	15	15	15	15	15	15	15	15	15	15
Number of communities surveyed	151 (100.6)	15 (100)	15 (100)	15 (100)	16 (106.7)	15 (100)	15 (100)	15 (100)	15 (100)	15 (100)	15 (100)
**Study participants**											
SAC targeted	4500	450	450	450	450	450	450	450	450	450	450
SAC recruited	4507 (100.1)	460 (102.2)	454 (100.9)	444 (98.7)	471 (104.7)	421 (93.6)	451 (100.2)	430 (95.6)	453 (100.7)	450 (100)	473 (105.1)
**Demography**											
Females	2272 (50.4)	221 (48)	251 (55)	235 (53)	234 (50)	191 (45)	241 (53)	223 (52)	232 (51)	225 (50)	219 (46)
Males	2235 (49.6)	239 (52)	203 (45)	209 (47)	237 (50)	230 (55)	210 (47)	207 (48)	221 (49)	225 (50)	254 (54)
Age [5–9 years]	2300 (51)	238 (52)	210 (46)	227 (51)	255 (54)	219 (52)	258 (57)	212 (49)	204 (45)	235 (52)	242 (51)
Age [10–14 years]	2207 (49)	222 (48)	244 (54)	217 (49)	216 (46)	202 (48)	193 (43)	218 (51)	249 (55)	215 (48)	231 (49)
**Samples collected**											
Number of SAC with stool *	4449 (98.7)	456 (99.1)	452 (99.6)	444 (100)	441 (93.6)	416(98.8)	447 (99.1)	430 (100)	453 (100)	450 (100)	459 (97.0)

PC: Preventive Chemotherapy; LGA: Local Government Area; AKN: Akure North; AKNW: Akoko Northwest; AKS: Akure South; AKSW: Akoko South West; IDA: Idanre; IFE: Ifedore; ODI: Odigbo; ONE: Ondo East; OSE: Ose; OWO: Owo; *: proportion of participants who returned specimen out of total recruited.

**Table 2:** Prevalence of soil-transmitted helminthiasis across LGAs and comparisons with the baseline survey.

			Impact Assessment								Baseline	Comparisons	
			*Ascaris lumbricoides*		Hookworm		*Trichuris trichiura*		Any STH		Any STH	Any STH	
		N	*n* (%)	95% CI	*n* (%)	95% CI	*n* (%)	95% CI	*n* (%)	95% CI	%	RR	d
Endemic LGAs with ≥ 5 effective rounds	AKSW	460	1 (0.2%)	−0.21, 0.64	1 (0.2%)	−0.21, 0.64	0 (0.0%)	-	2 (0.4%)	−0.17, 1.04	28.2	0.01	−99.9
	AKN	452	6 (1.3%)	0.27, 2.38	0 (0.0%)	-	1 (0.2%)	−0.21,0.65	7 (1.5%)	0.41, 2.68	39	0.04	−96.2
	IFE	444	8 (1.8%)	0.56, 3.04	2 (0.5%)	−0.17, 1.07	1 (0.2%)	−0.22, 0.67	11 (2.5%)	1.03, 3.92	25	0.10	−90.0
	ONE	471	27 (6.1%)	3.63, 7.83	10 (2.3%)	0.82, 3.43	1 (0.2%)	−0.20, 0.62	36 (8.2%)	5.24, 10.04	45.2	0.18	−81.9
Endemic LGAs with ≤ 5 effective rounds	AKS	421	3 (0.7%)	−0.09, 1.51	2 (0.5%)	−0.18, 1.13	0 (0.0%)	-	5 (1.2%)	0.15, 2.22	29	0.04	−95.9
	OSE	451	2 (0.4%)	−0.17, 1.05	8 (1.8%)	0.56, 2.99	0 (0.0%)	-	10 (2.2%)	0.86, 3.58	20	0.11	−89.0
	OWO	430	0 (0.0%)	-	0 (0.0%)	-	0 (0.0%)	-	0 (0.0%)	-	43	0.00	−100
	ODI	453	34 (7.5%)	5.08, 9.93	29 (6.4%)	4.15, 8.66	2 (0.4%)	−0.17, 1.05	58 (12.8%)	9.73, 15.9	38	0.34	−66.3
Low endemicity and no PC required	IDA	450	5 (1.1%)	0.14, 2.07	3 (0.7%)	−0.08, 1.41	0 (0.0%)	-	8 (1.8%)	0.55, 2.99	14.2	0.13	−87.3
	AKNW	473	2 (0.4%)	−0.16, 1.01	2 (0.4%)	−0.16, 1.01	0 (0.0%)	-	4 (0.9%)	0.02–1.67	5.2	0.17	−82.7

PC: Preventive Chemotherapy; LGA: Local Government Area; AKN: Akure North; AKNW: Akoko Northwest; AKS: Akure South; AKSW: Akoko South West; IDA: Idanre; IFE: Ifedore; ODI: Odigbo; ONE: Ondo East; OSE: Ose; OWO: Owo; RR: Risk Ratio; d: percentage decrease.

**Table 3:** Intensity of soil-transmitted helminthiasis infections across the LGAs.

		Endemic LGAs with ≥ 5 Effective Rounds				Endemic LGAs with ≤ 5 Effective Rounds				Low Endemicity and No PC Required	
		AKSW	AKN	IFE	ONE	AKS	OSE	OWO	ODI	IDA	AKNW
N	4507	460	454	444	471	421	451	430	453	450	473
***Ascaris lumbricoides***											
Negative	4364 (98.0%)	455 (99.8%)	446 (98.7%)	436 (98.2%)	414 (93.9%)	414 (99.3%)	446 (99.6%)	430 (100.0%)	419 (92.5%)	445 (98.9%)	459 (99.6%)
Light infection	88 (2.0%)	1 (0.2%)	6 (1.3%)	8 (1.8%)	27 (6.1%)	3 (0.7%)	2 (0.4%)	0 (0.0%)	34 (7.5%)	5 (1.1%)	2 (0.4%)
Moderate infection	0 (0.0%)	0 (0.0%)	0 (0.0%)	0 (0.0%)	0 (0.0%)	0 (0.0%)	0 (0.0%)	0 (0.0%)	0 (0.0%)	0 (0.0%)	0 (0.0%)
Heavy infection	0 (0.0%)	0 (0.0%)	0 (0.0%)	0 (0.0%)	0 (0.0%)	0 (0.0%)	0 (0.0%)	0 (0.0%)	0 (0.0%)	0 (0.0%)	0 (0.0%)
***Hookworms***											
Negative	4395 (98.7%)	455 (99.8%)	452 (100.0%)	442 (99.5%)	431 (97.7%)	415 (99.5%)	440 (98.2%)	430 (100.0%)	424 (93.6%)	447 (99.3%)	459 (99.6%)
Light infection	57 (1.3%)	1 (0.2%)	0 (0.0%)	2 (0.5%)	10 (2.3%)	2 (0.5%)	8 (1.8%)	0 (0.0%)	29 (6.4%)	3 (0.7%)	2 (0.4%)
Moderate infection	0 (0.0%)	0 (0.0%)	0 (0.0%)	0 (0.0%)	0 (0.0%)	0 (0.0%)	0 (0.0%)	0 (0.0%)	0 (0.0%)	0 (0.0%)	0 (0.0%)
Heavy infection	0 (0.0%)	0 (0.0%)	0 (0.0%)	0 (0.0%)	0 (0.0%)	0 (0.0%)	0 (0.0%)	0 (0.0%)	0 (0.0%)	0 (0.0%)	0 (0.0%)
***Trichuris trichiura***											
Negative	4447 (99.9%)	456 (100.0%)	451 (99.8%)	443 (99.8%)	440 (99.8%)	417 (100.0%)	448 (100.0%)	430 (100.0%)	451 (99.6%)	450 (100.0%)	461 (100.0%)
Light infection	5 (0.1%)	0 (0.0%)	1 (0.2%)	1 (0.2%)	1 (0.2%)	0 (0.0%)	0 (0.0%)	0 (0.0%)	2 (0.4%)	0 (0.0%)	0 (0.0%)
Moderate infection	0 (0.0%)	0 (0.0%)	0 (0.0%)	0 (0.0%)	0 (0.0%)	0 (0.0%)	0 (0.0%)	0 (0.0%)	0 (0.0%)	0 (0.0%)	0 (0.0%)
Heavy infection	0 (0.0%)	0 (0.0%)	0 (0.0%)	0 (0.0%)	0 (0.0%)	0 (0.0%)	0 (0.0%)	0 (0.0%)	0 (0.0%)	0 (0.0%)	0 (0.0%)

PC: Preventive Chemotherapy; LGA: Local Government Area; AKN: Akure North; AKNW: Akoko Northwest; AKS: Akure South; AKSW: Akoko South West; IDA: Idanre; IFE: Ifedore; ODI: Odigbo; ONE: Ondo East; OSE: Ose; OWO: Owo.

**Table 4:** Programmatic interpretation of STH prevalence data across ten LGAs.

		**LGA Categories at Baseline**		
Prevalence Thresholds	WHO Recommendations	Endemic LGAs with ≥ 5 Effective Rounds	Endemic LGAs with ≤ 5 Effective Rounds	Low Endemicity and No PC Required
0–1.9%	Conduct MDA at selected events and establish and maintain surveillance	Akoko South-west (0.4%); Akure North (1.5%)	Owo (0%); Akure South (1.2%)	Idanre (1.8%); Akoko Northwest (0.9%)
2–9.9%	Conduct MDA once in 2 years for 5 years and establish and maintain surveillance.	Ifedore (2.5%); Ondo East (8.2%)	Ose (2.2%)	None
10–20%	Conduct MDA once for 5 years and establish and maintain surveillance	None	Odigbo (12.8%)	None
>20%	Conduct MDA twice for 5 years and establish and maintain surveillance	None	None	None

PC: Preventive Chemotherapy.

**Table 5:** STH elimination insights across LGAs.

			Conditions That Must Be Met			
Category	LGAs	Baseline Prev.	Impact Prev.	MHI Prev.	Open Defecation *	MDA in WRA
		%	Prev. Must Be less than 20%	MHI Must Be < 2%	Should Be Significantly Low	Significant Reach and Coverage
Endemic LGAs with ≥ 5 effective rounds	Akoko South-west	28.2	0.4	0	24.1	No
	Akure North	39	1.5	0	29.1	No
	Ifedore	25	2.5	0	38.8	No
	Ondo East	45.2	8.2	0	47.8	No
Endemic LGAs with ≤ 5 effective rounds	Akure South	29	1.2	0	8.9	No
	Ose	20	2.2	0	44.2	No
	Owo	43	0	0	39.1	No
	Odigbo	38	12.8	0	21.0	No
Low endemicity and no PC required	Idanre	14.2	1.8	0	60.1	No
	Akoko North-west	5.2	0.9	0	43.7	No

PC: preventive chemotherapy; Prev: Prevalence; MHI: Moderate and heavy infections; WRA: Women of reproductive age; *: Open defecation was assessed via interview with each participant.

## Data Availability

The datasets used and analyzed during the current study can be obtained upon reasonable request from mogajihammed@gmail.com.
